# The Kashima Scan Study 2: a protocol for a prospective observational cohort study of cerebral small vessel disease in neurologically healthy adults

**DOI:** 10.1265/ehpm.25-00135

**Published:** 2025-07-03

**Authors:** Kohei Suzuyama, Yusuke Yakushiji, Akiko Matsumoto, Toshihiro Ide, Mikiko Tokiya, Atsushi Ogata, Junko Nakajima, Tatsumi Hirotsu, Shuhei Ikeda, Tatsuya Doyama, Masayasu Morikawa, Yuta Goto, Yoshiko Katsuki, Kazuhiro Kawamoto, Yoshimasa Oda, Haruki Koike, Hideo Hara

**Affiliations:** 1 Division of Neurology, Department of Internal Medicine, Faculty of Medicine, Saga University, Saga, Japan; 2 Department of Neurology, Kansai Medical University, Hirakata, Japan; 3 Department of Social Medicine, Saga University, Saga, Japan; 4 Department of Neurosurgery, Saga University Faculty of Medicine, Saga, Japan; 5 Department of Radiology, Oda Hospital, Saga, Japan; 6 Department of Neurosurgery, Oda Hospital, Saga, Japan; 7 Department of Internal Medicine, Oda Hospital, Saga, Japan

**Keywords:** Small vessel disease, Stroke, Cardiovascular disease, Cognitive impairment, Genetic polymorphism, Lifestyles

## Abstract

**Background:**

Our previous observational cohort study, the Kashima Scan Study (KSS), identified associations between lifestyle, cerebral small vessel disease (SVD) as detected by magnetic resonance imaging of the brain, and disease outcomes including cognitive impairment and vascular diseases. However, established modifiers of the outcomes such as genetic background, drinking and exercise habits, and socioeconomic status were not considered. Regarding genetic factors in particular, the *ALDH2* rs671 variant, East Asian-specific diversity, and *APOE* status are expected to have strong effects. The aim of KSS-2 is to examine the interactions of genetic background, lifestyle factors including drinking habit, socioeconomic status, and/or SVD markers for cognitive impairment, vascular disease, and death.

**Method:**

The KSS-2 is a prospective regional observational study of a healthy Japanese cohort that will clarify lifestyle habits to better maintain brain health from midlife by genotype. Japanese adults who underwent brain health checkups at their own expense are enrolled and will be followed-up for 10 years. We will extend the protocol of the KSS to include genetic background and potential confounding factors, including lifestyle (including drinking and exercise habit) and socioeconomic status, and perform survival analyses. The study outcomes are cognitive impairment, vascular events, and death.

**Results:**

We enrolled 908 healthy adults (mean age 64.2 years; range 35 to 84 years; 41% male) from September 1, 2018 until December 31, 2024.

**Conclusion:**

This study will provide important insights into the development of individualized health intervention strategies.

**Supplementary information:**

The online version contains supplementary material available at https://doi.org/10.1265/ehpm.25-00135.

## Graphical Abstract

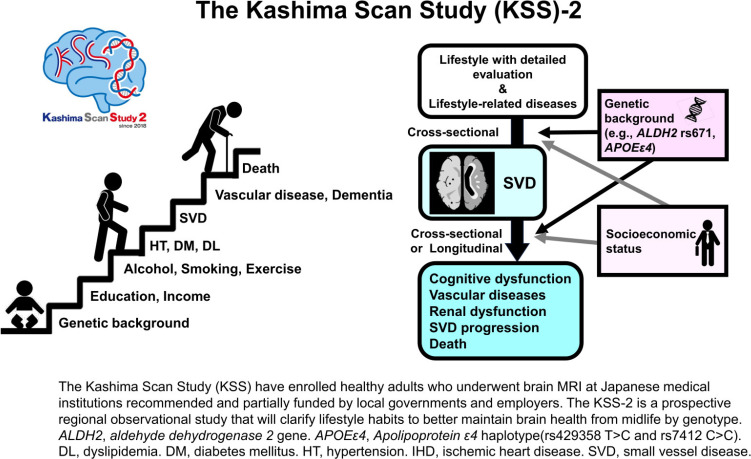

## Introduction

### Background

Preventive medicine for healthy individuals requires early detection of disease and consideration of the interactions between multiple risk factors, including genetic polymorphisms. Cerebral small vessel disease (SVD) caused by damage to the small vessels, such as capillaries and meningeal arteries [1], is clinically recognizable as residual lesions (i.e., SVD markers) on brain imaging even before any neurological symptoms develop. The main causes of SVD are hypertensive arteriosclerosis (i.e., type 1 SVD) and cerebral amyloid angiopathy (i.e., type 2 SVD) [2], which are major pathogenic factors for stroke [3] and dementia [4]. Lacunae, cerebral microbleeds (CMBs), white matter hyperintensities (WMH), periventricular hyperintensity (PVH), perivascular spaces (PVS), cortical superficial siderosis (cSS), and a comprehensive score system based on these SVD markers (i.e., summary SVD score) [5, 6] can be evaluated from brain MRI, allowing estimation of the main pathogenic pathways of SVD in individuals. Identification of factors associated with individual SVD markers, as well as the summary SVD score, will provide important insights into the prevention and management of stroke and dementia.

We have been continuing an observational cohort study, designated the Kashima Scan Study (KSS), which was established in 2005. We demonstrated several associations with SVD in healthy Japanese adults who underwent health screening tests involving brain MRI. For example, this cohort first reported that CMBs, particularly in the deep or infratentorial regions, are associated with cognitive impairment in healthy adults [7, 8]. We also demonstrated that a summary SVD score (i.e., total SVD score) is predictive of subsequent cerebro-cardiovascular events [9].

**Fig. 1:**
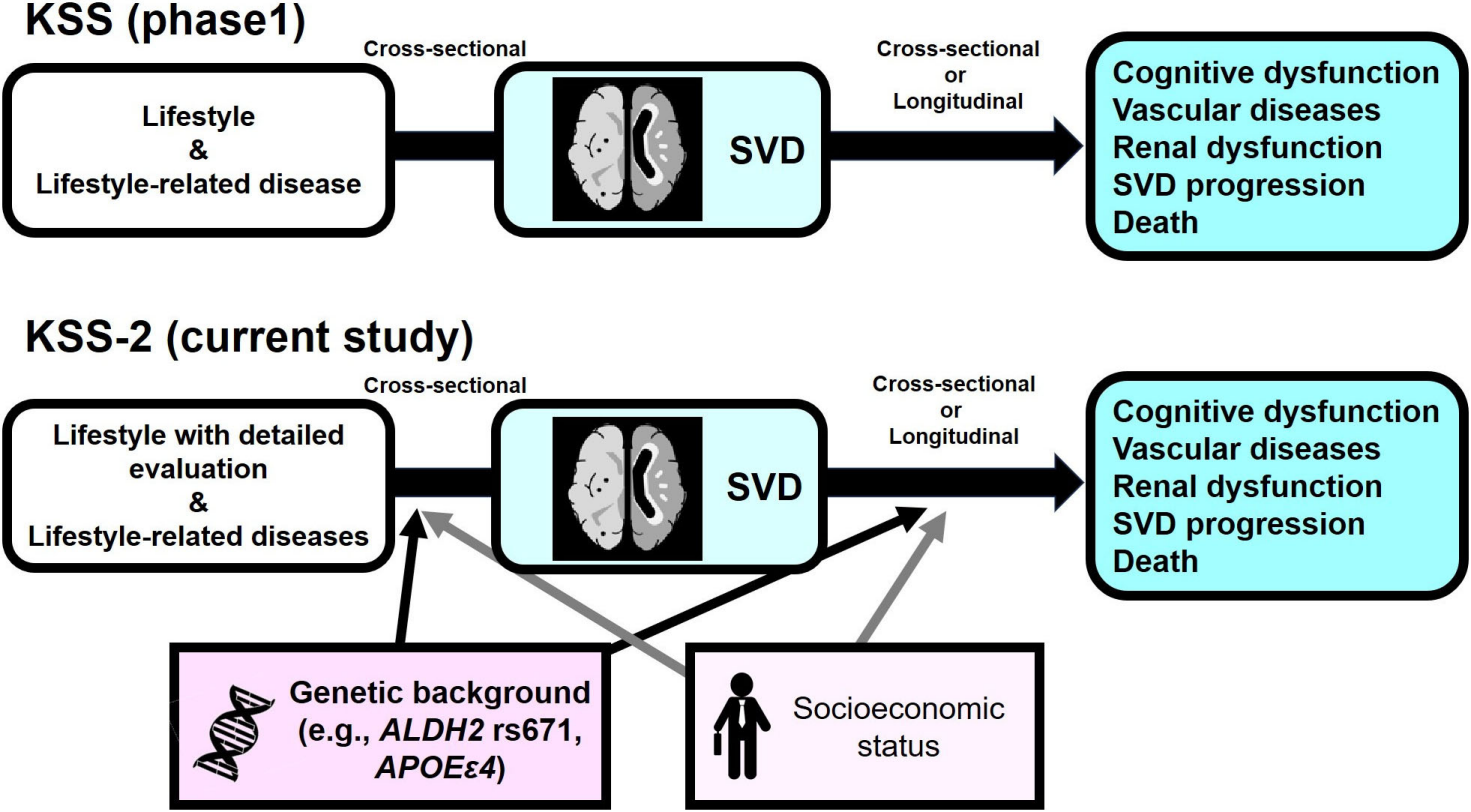
Overview of the Kashima Scan Studies The Kashima Scan Study (KSS) (upper panel) enrolled 1738 participants who underwent brain MRI at Japanese medical institutions between 2005 and 2011, recommended and partially funded by local governments and employers. The KSS reported associations between lifestyle and lifestyle-related disease, cerebral small vessel disease (SVD) as diagnosed by MRI, and disease outcomes. The current protocol for the KSS-2 (lower panel), enrolling patients from 2018 to 2024, extended the approach to include genetic background, lifestyle (including drinking and exercise habits) and socioeconomic status, as established factors influencing outcomes. *ALDH2*, aldehyde dehydrogenase 2 gene; *APOEε4*, apolipoprotein ε4 haplotype (rs429358 T>C and rs7412 C>C).

In this new study, the Kashima Scan Study 2 (KSS-2), we plan to incorporate factors for which associations with SVD in the general population (particularly Japanese people) is not fully understood. Those factors include genetic polymorphisms [10, 11] drinking habit, and socioeconomic status. Regarding genetic polymorphisms and drinking habit, we paid special attention to aldehyde dehydrogenase 2 (*ALDH2*, rs671) and alcohol dehydrogenase 1B (*ADH1B*, rs1229984) which are involved in alcohol metabolism. The *ALDH2* gene is associated with not only drinking habit [12], but also various diseases, including malignant neoplasms [13], vasospastic angina [14], stroke [15, 16] and dementia [17]. Previous studies reported rare *NOTCH3* variants and rs12204590 variant (near *FOXF2*) are related with WMH burden [18, 19]. *ALDH2* has been reported to be associated with CMBs [11] and apolipoprotein E (*APOE*) has been shown to be associated with strictly lobar microbleeds [10] and cSS [20], but few reports have investigated links to SVD markers among healthy populations. Socioeconomic status is accepted as a major modifiable risk factor for vascular diseases and dementia, but little is known regarding whether this factor could be independently associated with SVD [21]. The aim of the KSS-2 is thus to clarify the independent or synergistic influences of those genotypes, lifestyle factors including drinking habit, socioeconomic status, and/or SVD markers on cognitive impairment, vascular disease, renal dysfunction, SVD progression, and death in healthy Japanese adults in midlife (Fig. 1).

## Methods

### Design

The KSS-2 is a prospective, regional observational cohort study of neurologically healthy adults that will be reported with reference to the Strengthening the Reporting of Observational Studies in Epidemiology guidelines [22]. Subjects have been recruited among individuals who have undergone health screenings involving brain MRI in our center (Yuai-Kai Oda Hospital) at their own expense (in many cases, partial financial support is provided by local governments or the companies they work for) since September 1, 2018. Enrolment of subjects was finished on December 31, 2024. We enrolled 908 healthy adults (mean age 64.2 years; range 35 to 84 years; 41% male). Subjects will be followed-up for 10 years after enrolment.

### Participant population - inclusion and exclusion criteria

Inclusion and exclusion criteria for participant in the KSS-2 are similar to those of the KSS [8]. Inclusion criteria are: age ≥20 years; no disability in instrumental activities of daily living; and provision of written informed consent. Exclusion criteria are: inability to undergo brain MRI; history of traumatic brain injury or neurological disorder; history of ischemic heart disease; or neurological abnormalities on examination.

### Baseline assessment

Details of the baseline assessments are shown in the Table 1. All subjects are first examined by a general physician or certified neurosurgeon. Subjects with suspected neurological deficits or abnormal findings on MRI subsequently undergo neurological examination by a certified neurologist. The clinical features recorded are essentially the same as those in the previous KSS: age, sex, body mass index, smoking status, history of ischemic heart disease, duration of education, actual blood pressure (BP) values, and presence of hypertension, diabetes mellitus, and dyslipidemia. The definitions used to identify hypertension, diabetes mellitus, and dyslipidemia are similar to the previous KSS (Table S1). Subjects who are smokers at baseline are classified as current smokers. Brinkman index is calculated using the questionnaire (Table S2). History of ischemic heart disease and educational background are elicited in interviews with each subject. In addition, in the KSS-2 we also record information on alcohol intake, exercise habit, occupation, and income collected from questionnaires [Tables S2 and S3], as well as findings of atrial fibrillation or cardiac hypertrophy on electrocardiography. Regarding cognitive evaluation in the KSS-2, the Japanese version of the Montreal Cognitive Assessment (MoCA-J) is administered by a skilled nurse.

**Table 1:** Study protocol of the Kashima Scan Study 2

	Baseline	2^nd^ year	4^th^ year	…	10^th^ year
Informed consent	X				
Clinical assessment	X				
Brain MRI	X				
MoCA-J	X				
Blood and urine test, ECG	X				
Gene tests (*ALDH2*, *ADH1B*, *APOE*)	X				
Questionnaire* about cerebro-cardiovascular events		X	X	…	X
Questionnaire about MCI status					X

ADH1B, alcohol dehydrogenase 1B gene; ALDH2, aldehyde dehydrogenase 2 gene; APOE, apolipoprotein E gene; ECG, electrocardiogram; MCI, mild cognitive impairment; MoCA-J, Japanese version of the Montreal Cognitive Assessment; MRI, magnetic resonance imaging.

*Participants will be divided into two groups, with half of the group receiving surveys in odd-numbered years and the other half receiving surveys in even-numbered years, meaning that each participant will be surveyed every two years about events that occurred over the preceding two years.

### Evaluation of MRI findings

All brain MRI will be performed using a 1.5-T scanner. We will evaluate the following SVD markers on MRI: lacunae, CMBs, WMH, PVH, cSS, and brain atrophy. Individual SVD markers will be evaluated based on validated rating methods and a scoring system [6]. Lacunae are defined as focal, sharply demarcated lesions 3 to 15 mm in diameter showing high intensity on T2 weighted image (WI) and low intensity on T1WI. CMBs is defined as rounded areas of signal loss on T2* Gradient Echo, 2–10 mm in diameter. The flow void artifacts of pial vessels and symmetrical hypointensities in the globus pallidum caused by calcification are excluded. Severity of WMH or PVH on T2WI and Fluid Attenuated Inversion Recovery imaging is rated according to the Fazekas scale (WMH: grade 1, punctuate; grade 2, early confluence; and grade 3, confluent; PVH: grade 1, caps or lining; grade 2, bands; and grade 3, irregular extension into deep white matter) [2]. PVS is rated on axial T2-weighted MRI by a validated visual rating scale. PVS is defined as small, sharply delineated structures with the same or very similar signal intensity as CSF, measuring 3 mm and following the course of perforating or medullary vessels. PVS is assessed in basal ganglia and centrum semiovale (CSO) regions. For both areas, PVS rating scores is as follows: 0 = no PVS; 1 = 1–10 PVS; 2 = 11–20 PVS; 3 = 21–40 PVS; and 4 = 40 or more PVS. The numbers refer to PVS on one side of the brain: after reviewing all relevant slices for the anatomical area being assessed, scores for the slice and side with the highest number of PVS is recorded. The definition of cSS is chronic blood products on or overlying the superficial cortex. The severity and distribution of cSS is classified as focal (restricted to ≤3 sulci) or disseminated (≥4 sulci) [6, 23].

Regarding summary SVD burden, total SVD score will then be also assessed [5]. It ranges from 0 to 4, evaluated based on a previously developed scoring system. The presence of lacunae or CMBs is defined as the presence of one or more foci (1 point each); presence of moderate to severe WMH or PVH is defined as either confluent deep WMH (Fazekas score 2 or 3) and/or irregular PVH extending into deep white matter (Fazekas score 3) (1 point); and presence of moderate to severe PVS (1 point). To calculate total CAA MRI SVD score, ranging from 0 to 6 points, we will also evaluate lobar CMBs, cSS, CSO-PVS and WMH. The rating scale of lobar CMBs is as follows: 1 = 2–4 CMBs; 2 = ≥5 CMBs. The cSS score is 1 point if focal and 2 points if disseminated. The CSO-PVS is 1 point for moderate-to-severe PVS (grade 3 or 4). The WMH score is 1 point if Fazekas score is ≥2 [23].

Individual SVD markers will be assessed by certified stroke neurologists. Inter-rater reliability values for these variables will be expressed using Cohen’s kappa value. Further details of the specific protocols are provided in Table S4.

### Biomaterials

Peripheral blood samples for blood testing and a spot urine specimen for urine testing are collected at the time of the health screening examination. The remaining serum (approximately 6 mL) is frozen and stored in the Division of Neurology, Department of Internal Medicine, Saga University. The urine test includes testing for urine sugar and protein levels.

### Genetic examinations

We evaluate polymorphisms in *ALDH2*, *ADH1B* and *APOE* using DNA extracted from buccal mucosa by real-time PCR. Buccal swabs will be collected and immersed in distilled water. The precipitate obtained by removing supernatant after centrifugation will be dissolved in Direct PCR Lysis Reagent (VIAGEN Biotech, Los Angeles, CA, USA) with proteinase K, incubated at 55 °C overnight, then at 85 °C for 45 minutes, and used as the PCR template. TaqMan^®^ SNP genotyping assays will be performed with a mixed reagent consisting of primer and probe (C__11703892_10, C___3084793_20, C___904973_10, for ALDH2 rs671 (G>A), ADH1B rs1229984 (A>G), APOE rs429358 (T>C) and rs7412 (C>T)) according to the protocol provided by the manufacturer (Thermo Fisher Scientific, Waltham, MA, USA). The APOE genotype will be determined as indicated in Table S5. The DNA obtained from participants can be used for secondary purposes.

### Follow-up schedule

Subjects will be followed-up for 10 years from the entry. To obtain information about cerebro-cardiovascular events and all-cause death, we will send out a survey every two years (Table S6) to all subjects (Table 1). We define cerebro-cardiovascular events as cerebrovascular events (i.e., cerebral infarction, transient ischemic attack [TIA; defined as a temporary neurological deficit resolving within 24 h without apparent abnormalities on diffusion-weighted imaging], cerebral hemorrhage, or subarachnoid hemorrhage managed with hospitalization) and cardiovascular events (i.e., myocardial infarction, angina pectoris, aortic dissection, or acute heart failure managed with hospitalization). When any events are reported, the authors will visit the hospitals that treated the participant and obtain the medical records. Subjects with incomplete information from follow-up questionnaires are excluded. The period of follow-up is estimated based on the period for which valid responses are received from subjects. Ten years after entry, questionnaires regarding cognitive decline (such as Informant Questionnaire on Cognitive Decline for the Elderly) will be sent to family members, or telephone interviews will be conducted with the participant, their family, and their primary care physician to examine for transitions to mild cognitive impairment or dementia.

### Primary outcomes

Cognitive impairment, cerebral cardiovascular disease, and death

### Secondary outcomes

Progression of SVD and impaired renal function

### Sample size estimates

We have previously reported evidence from a cross-sectional study of a subset of KSS participants (N = 518, mean age 56.1 years; range 33 to 85 years; including 35 participants with CMBs) that the odds ratio of mild cognitive impairment (Mini Mental State Examination score < 27) for CMBs (explanatory variable) was 5.44 [7]. Based on that study, the sample size sufficient to detect a similar association in KSS-2 is 518 with power of 0.9 and an alpha error of 0.05. In addition, we reported a survival analysis for 1349 participants (mean age 57.7 years; range 22 to 85 years) in the KSS, including 96 participants with baseline SVD ≥2. After a mean follow-up of 6.7 years, 35 cerebrovascular events occurred [9], giving a sample size estimate of 620 with 5 years of follow-up, power of 0.9 and an alpha error of 0.05. Given the dropout rate of 9.0%, 690 would be needed to reproduce the association. Age is critical in determining sample size because of its effect on event occurrence, and we expected a similar age distribution because KSS-2 was conducted at the same institution as the previous KSS, but the average age of participants in KSS-2 was approximately 6 to 8 years higher, so more events are expected. On the other hand, the interactive effect of ALDH2 and APOE genes has not been reported, making it difficult to determine the required sample size. Given this situation, we set a maximum achievable sample size of 850–900.

### Statistical analysis

Statistical analyses will be performed using SAS software (version 9.4; SAS Institute, Cary, NC, USA). Univariate analyses will be performed using Fisher’s exact test, t-test, Mann–Whitney U test, and analysis of variance, as appropriate. Survival analyses will be primarily performed using the Kaplan–Meier method and Cox proportional hazards modeling. Explanatory variables will focus on the SVD effect and the interactive effect of SVD and genetic background (rs671, rs1229984, rs429358, rs7412). In addition, interactions between lifestyle and SVD or genetic background will be considered to generate evidence that can contribute to individualized disease prevention. Cross-sectional and stratified analyses will be performed as appropriate. In addition, association analysis between variables will be performed using methods including structural equation modeling.

### Study organization

The KSS is organized by Saga University and Kansai Medical University (KMU).

## Discussion

The KSS-2 will determine how the genotypes (e.g., *ALDH2*, *ADH1B*, and *APOE*), SVD burden, and/or lifestyle factors interact with cognitive impairment, cerebral cardiovascular disease, and death. In secondary analysis, we will assess whether the targeted genotypes, SVD burden, and/or lifestyle factors interact with progression of SVD and impaired renal function.

A previous study reported differences in anatomical distributions of CMBs between eastern and western populations. Eastern Asian populations had higher prevalence of deep and infratentorial or mixed CMBs, implying differences in age, hypertension, or lifestyle-related SVD (i.e., type 1 SVD) among different ethnicites [24]. The defective allele of the aldehyde dehydrogenase 2 gene, ALDH2*2, has a higher frequency in East Asia than in western countries [25]. Almost half of Japanese carries ALDH2*2 [26]. The KSS-2 will explore the causes of differences in sporadic SVD pathologies between ethnicities from genetic and East Asian lifestyle or cultural perspectives.

## Conclusion

The KSS-2 is a prospective regional observational study of a healthy Japanese cohort that will clarify lifestyle habits to better maintain brain health from midlife by genotype.

## Abbreviations

ADH1B: alcohol dehydrogenase 1B
ALDH2: aldehyde dehydrogenase 2
APOE: apolipoprotein E
CMBs: cerebral microbleeds
cSS: cortical superficial siderosis
KSS: the Kashima Scan Study
MoCA-J: Japanese version of the Montreal Cognitive Assessment
PVH: periventricular hyperintensities
PVS: perivascular spaces
SVD: cerebral small vessel disease
WMH: white matter hyperintensities

## Supplementary information

**Additional file 1:** **Table S1.** Definitions of hypertension, diabetes mellitus and dyslipidemia. **Table S2.** Questionnaire for smoking and drinking habits. **Table S3.** Questionnaire at health screening test. **Table S4.** Settings for magnetic resonance imaging (MRI) of the brain. **Table S5.** The determination of APOE genotype. **Table S6.** Annual questionnaire for follow-up.

## Declarations

### Availability of data and materials

The data that support the findings of this study is available from the corresponding author upon reasonable request.
