# Supplementary material for: The Kashima Scan Study 2: a protocol for a prospective observational cohort study of cerebral small vessel disease in neurologically healthy adults
Source: Environ Health Prev Med. 2025 Jul 3;30:52. doi: 10.1265/ehpm.25-00135 (PMC12256151; doi:10.1265/ehpm.25-00135)
Supplement: Supplementary file 1 — Additional file 1: Table S1. Definitions of hypertension, diabetes mellitus and dyslipidemia. Table S2. Questionnaire for smoking and drinking habits. Table S3. Questionnaire at health screening test. Table S4. Settings for magnetic resonance imaging (MRI) of the brain. Table S5. The determination of APOE genotype. Table S6. Annual questionnaire for follow-up. [file ehpm-30-052-s001.docx]

**Table S1: Definitions of hypertension, diabetes mellitus and dyslipidemia**

|  | Definition |
| --- | --- |
| Hypertension | Systolic BP ≥140 mmHg and/or diastolic BP ≥90 mmHg or administration of antihypertensive agents |
| Diabetes mellitus | Hemoglobin A1c level ≥6.5% and/or fasting serum glucose level ≥126 mg/dL and/or administration of anti-diabetic agents |
| Dyslipidemia | Fasting serum low-density lipoprotein cholesterol ≥140 mg/dL and/or high-density lipoprotein cholesterol <40 mg/dL and/or triglycerides ≥150 mg/dL and/or administration of anti-dyslipidemic agents |

**Table S2: Questionnaire for smoking and drinking habits**

| 1 | How many cigarettes do you smoke a day on average? |
| --- | --- |
|  | I have never smoked. |
|  |  |
|  | I don’t smoke now, but I used to. |
|  | From the age of ___ to ___, I smoked ___ cigarettes a day on average. |
|  | From the age of ___ to now, I have smoked ___ cigarettes a day on average. |
|  |  |
| 2 | How often do you consume alcohol? |
|  | I have never consumed alcohol. (You do not have to answer questions 5 and 6) |
|  | I consume alcohol less than once a month. (You do not have to answer questions 5 and 6) |
|  |  |
|  | Once a month |
|  | Twice a month |
|  | Three days a month |
|  | Once a week |
|  | Twice a week |
|  | Three days a week |
|  | Four days a week |
|  | Five days a week |
|  | Six days a week |
|  | Daily |
|  |  |
| 3 | How long have you had a habit of drinking more than once a week? |
|  | Never |
|  | Less than 10 years |
|  | 10 to 20 years |
|  | 20 to 30 years |
|  | 30 to 40 years |
|  | 40 to 50 years |
|  | More than 50 years |
|  |  |
| 4 | Have you ever had a habit of drinking? |
|  | □ Yes □ No |
|  | If you have had a habit of drinking, but don’t drink now, please answer the question below. |
|  | From age of ___ to ___, I drank _____ (kind of alcohol) _____ (amount) a day, ____ times a week. |
|  | Why did you quit drinking? |
|  |  |
| 5 | How much alcohol do you drink? |
|  | Please choose the category of alcohol you usually consume and indicate the amount you drink daily. |
|  |  |
|  | Beer (alcohol by volume: 5%) |
|  | Large bottle (633 mL): bottle(s)/day |
|  | Medium bottle (500 mL): bottle(s)/day |
|  | Large can (500 mL): can(s)/day |
|  | Medium can (350 mL): can(s)/day |
|  | Mug (633 mL): mug(s)/day |
|  | Cup (180 mL): cup(s)/day |
|  |  |
|  | Sake (alcohol concentration: 15%) |
|  | Cup: gou/day |
|  | Ochoko (30 mL) : ochoko/day |
|  |  |
|  | Shochu (alcohol concentration 25%) |
|  | Straight (180 mL: cup(s)/day |
|  | Shochu 3: hot water 7 cup(s)/day |
|  | Shochu 6: hot water 4 cup(s)/day |
|  | Shochu 8: hot water 2 cup(s)/day |
|  | Can (8%) (350 mL): can(s)/day |
|  | Can (4%) (350 mL): can(s)/day |
|  |  |
|  | Whisky, brandy (alcohol concentration: 43%) |
|  | Bottle (750 mL): bottle(s)/day |
|  | On the rocks: glass(es)/day |
|  | Double: glass(es)/day |
|  | Single: glass(es)/day |
|  |  |
|  | Wine (alcohol concentration: 12%) |
|  | Wine glass (120 mL): glass(es)/day |
|  |  |
|  | Other |
|  | Kind of alcohol : /day |
|  |  |
| 6 | How often do you drink more than 3 cans of beer or 2.7 gou of sake a day (or equivalent amount of alcohol)? |
|  | Never |
|  | Less than once a month |
|  | Once a month |
|  | Once a week |
|  | Every day or almost every day |

**Table S3: Questionnaire at health screening test**

|  | Socioeconomic background |
| --- | --- |
| 1 | What is your final academic background? |
| □  □  □  □  □  □  □  □ | Ordinary elementary school  Higher elementary school  Elementary school  Junior high school  High school  Professional training college ( years)  College ( years)  Graduate school ( years) |
|  | → I studied at school for a total of years. |
|  |  |
| 2 | What is the longest job you have ever held? |
|  | ( ) |
|  |  |
| 3 | Please classify that occupation using one of the following categories. |
| □  □  □  □ | Clerical work  Agriculture, forestry and fisheries industry  Self-employed  Other ( ) |
|  |  |
| 4 | What is the highest annual income in your household? |
| □  □  □  □  □  □  □  □  □  □  □ | 0-999,999 yen  1000,000-1999,999 yen  2000,000-2999,999 yen  3000,000-3999,999 yen  4000,000-4999,999 yen  5000,000-5999,999 yen  6000,000-6999,999 yen  7000,000-7999,999 yen  8000,000-8999,999 yen  9000,000-9999,999 yen  10,000,000 yen or more |
|  | Exercise habits |
| 1 | Do you engage in regular exercise? |
| □  □ | Yes → Please answer the questions below  No |
| 2 | What is the most frequent exercise you engage in? |
| □  □  □ | Walking → □ slow, □ normal, □ fast  Jogging → □ slow, □ normal, □ fast  Other ( ) |
|  | How many hours per week do you engage in this exercise?  → 　　　　 hour(s)/week  How many months per year do you engage in this exercise?  → 　　　　 month(s)/year |
| 3 | What is the second most frequent exercise you engage in? |
| □  □  □ | Walking → □ slow, □ normal, □ fast  Jogging → □ slow, □ normal, □ fast  Other ( ) |
|  | How many hours per week do you engage in this exercise?  → 　　　　 hour(s)/week  How many months per year do you engage in this exercise?  → 　　　　 month(s)/year |
|  |  |
|  | Thank you for completing our questionnaire. |

**Table S4: Settings for magnetic resonance imaging (MRI) of the brain**

- *Period 1 (from September 1, 2018 to January 31, 2023)*

EXCELART Vantage, version 7.0; Toshiba Medical Systems, Tokyo, Japan

- *Period 2 (from February 1, 2023)*

VANTAGE FORTIAN MRT-1550; CANON Medical Systems, Tokyo, Japan

|  | T2 | | T1 | | FLAIR | | Ｔ2^*^ | | DWI | | MRA | |
| --- | --- | --- | --- | --- | --- | --- | --- | --- | --- | --- | --- | --- |
| Period | 1 | 2 | 1 | 2 | 1 | 2 | 1 | 2 | 1 | 2 | 1 | 2 |
| Repetition time (ms) | 4950 | 4000 | 2250 | 500 | 10,000 | 10,000 | 515 | 465 | 4900 | 7000 | 20 | 32 |
| Echo time (ms) | 84 | 108 | 10 | 15 | 123.5 | 250 | 13.5 | 20 | 90 | 130 | 6.8 | 6.8 |
| Number of excitations | 1 | | 1 | | 1 | | 1 | | 2 | | 1 | |
| Flip angle | 90 | | 90 | 90 | 90 | 90 | 20 | 30 | 90 | 90 | 16 | 20 |
| Slice (mm) | 5 | 7 | 5 | 7 | 5 | 7 | 5 | 7 | 5 | 7 | 0.8 | 1 |
| Gap (mm) | 1 | 1.4 | 1 | 1.4 | 1 | 1.4 | 1 | 1.4 | 1 | 1.4 |  |  |
| Matrix (mm^2^) | 352*352 | 352*400 | 256*320 | 256*352 | 256*320 | 224*336 | 224*352 | 224*320 | 160*160 | 160*160 | 224*352 | 176*272 |
| Field of view (mm^2^) | 22*22 | | 22*22 | | 22*22 | | 22*22 | | 22*22 | 26*28 | 19*20 | 0 |

**Table S5: The determination of APOE genotype**

| rs429358 | rs7412 | APOE allele |
| --- | --- | --- |
| T/T | C/C | ε3/ε3 |
| T/C | C/C | ε4/ε3 |
| C/C | C/C | ε4/ε4 |
| T/T | C/T | ε2/ε3 |
| C/T | C/T | ε2/ε4 |

**Table S6: Annual questionnaire for follow-up**

|  | **Question 1** |
| --- | --- |
|  | We would like you to ask whether you received treatment for any of the diseases listed below while in hospital. If an abnormality was unexpectedly found on imaging (i.e., CT or MRI) without any symptoms, please do not check “yes”.  From your health screening test to March 31st, 20** |
|  | Stroke |
|  | Cerebral infarction: Yes / No  Brain hemorrhage: Yes / No  Subarachnoid hemorrhage: Yes / No  Transient ischemic attack: Yes / No  I had a stroke, but I do not know what kind: Yes / No |
|  | Cardiovascular disease managed with hospitalization |
|  | Angina pectoris: Yes / No  Myocardial infarction: Yes / No  Aortic dissection: Yes / No  Acute heart failure: Yes / No  Cardiovascular disease, but I do not know which kind: Yes / No |
|  |  |
|  | **Question 2** |
|  | If you checked “yes” above, we would like you to provide the date or hospital in which you had a stroke.  If you were hospitalized multiple times, please describe all events. |
|  | Stroke |
|  | When did you have a stroke?  Which hospital did you visit? |
|  |  |
|  | Cardiovascular disease managed with hospitalization |
|  | When did you develop cardiovascular disease?  Which hospital did you visit? |
|  | Thank you for completing our questionnaire. |
